# Synchronous Versus Metachronous Multiple Malignant Tumors Involving the Digestive Tract: Predictors of Survival from a Single-Center Retrospective Study

**DOI:** 10.3390/medicina61111962

**Published:** 2025-10-31

**Authors:** Alexandru Vlad Oprita, Cornelia Nitipir, Eduard Achim, Florin Andrei Grama

**Affiliations:** 1 Department of Oncology, Faculty of Medicine, Carol Davila University of Medicine and Pharmacy, 050474 Bucharest, Romania; alexandruvladoprita@yahoo.com (A.V.O.); cornelia.nitipir@umfcd.ro (C.N.); 2 Department of Medical Oncology, “Saint Nicholas” Hospital Pitești, 110124 Pitești, Romania; 3 Department of Medical Oncology, Agrippa Emergency Clinical Hospital, 011356 Bucharest, Romania; 4 Faculty of Medicine, “Iuliu Hațieganu” University of Medicine and Pharmacy, 400012 Cluj-Napoca, Romania; 5 Coltea Clinical Hospital, Ion C. Brătianu Boulevard 1, 030167 Bucharest, Romania; florin.grama@umfcd.ro; 6 Department of Surgery, Carol Davila University of Medicine and Pharmacy, 050474 Bucharest, Romania

**Keywords:** multiple primary malignant tumors, synchronous, metachronous, digestive tract, survival, restricted mean survival time, single center

## Abstract

*Background*: Multiple primary malignant tumors (MPMTs) involving the digestive tract pose diagnostic and therapeutic challenges, with survival differences between synchronous and metachronous forms not well defined. This study assessed predictors of overall survival (OS) in patients in whom at least one tumor originated in the digestive tract or accessory organs. *Methods*: We retrospectively reviewed 1920 oncology cases (January 2020–June 2023) from St. Nicholas Hospital, Romania. Of 118 patients with MPMTs, 45 had ≥1 digestive tract tumor. They were classified as synchronous (<2 months) or metachronous (>2 months) as per the SEER rules. Clinical, pathological, treatment, and follow-up data were analyzed; OS was evaluated using Kaplan–Meier and Cox regression. *Results*: Fifteen patients (33%) had synchronous tumors and 30 (67%) had metachronous tumors. Overall, 17 of 45 patients (37.8%) died by the last follow-up. The restricted mean survival time (RMST) was 31.3 months for those with synchronous vs. 68.3 months for those with metachronous tumors (HR = 2.49, 95% CI 0.95–6.50, *p* = 0.062; log-rank *p* = 0.053). Curative treatment of the first tumor was associated with markedly improved survival (RMST 58.2 vs. 29.4 months; HR = 20.5, 95% CI 3.68–114, *p* < 0.001). In the multivariable Cox regression analysis, advanced primary nodal stage (N2–N3) remained independently associated with reduced survival (adjusted HR 3.86, 95% CI 1.04–14.3, *p* = 0.044). The adjusted effect of synchronous vs. metachronous classification was attenuated (adjusted HR 2.22, 95% CI 0.84–5.86, *p* = 0.10). *Conclusions*: In this single-center, hypothesis-generating cohort, synchronous digestive-tract MPMTs were associated with shorter unadjusted survival than metachronous tumors, but advanced nodal stage and limited feasibility of curative therapy were the dominant independent predictors of poor outcome. Given the small sample size and retrospective design, these findings should be interpreted as preliminary and warrant validation in larger, multicenter cohorts.

## 1. Introduction

Multiple primary malignant tumors (MPMTs) are increasingly observed in contemporary oncology, reflecting advances in diagnostic imaging, expanded screening programs, and improved cancer-specific survival. Additional contributors include environmental exposures, infectious agents, inherited genetic predispositions, and the long-term effects of prior therapies. As patients live longer after an initial cancer diagnosis, the risk of developing subsequent primary tumors rises [1].

Definitions of MPMTs vary across registries. The International Association of Cancer Registries/International Agency for Research on Cancer (IACR/IARC) considers the colon a single site, whereas the Surveillance, Epidemiology, and End Results (SEER) program distinguishes tumors arising in different segments. Similarly, synchronous tumors are defined as occurring within six months by IACR/IARC, compared to a two-month window in the SEER program. Awareness of these differences is essential when interpreting epidemiological data [2,3].

The reported incidence of MPMTs ranges from 2.4% to 17.2% [4], though single-center studies, such as in Chinese populations, report lower prevalences (~1%) [5]. Secondary primaries most frequently emerge within the first 1–5 years after the index malignancy, with a smaller but distinct rise at 10–15 years. A considerable proportion of about 12.6% arise in the same organ, most often involving the breast or lung [6]. Certain tumor combinations are particularly common. For instance, head and neck cancers often coincide metachronously with esophageal tumors, while prostate and colorectal cancers frequently occur together. Uterine and ovarian malignancies show a strong synchronous association [7]. The overall leading associations are thought to involve digestive tumors [8,9], while patients assigned female at birth with breast cancer have a higher probability of developing another primary gynecologic cancer [10]. These combinations are influenced by shared genetic predispositions (such as BRCA mutations for breast-gynecologic cancers), environmental exposures, and field cancerization effects, particularly in the aerodigestive tract. Clinical implications include the need for tailored surveillance strategies and heightened suspicion for additional primaries in patients with these index cancers.

Prognosis is generally poorer for synchronous compared with metachronous MPMTs as synchronous tumors often present at more advanced stages and require more complex management. Digestive system MPMTs carry some of the worst outcomes. In metachronous cases, shorter intervals between tumor diagnoses (≤60 months) correlate with reduced survival [8].

Accurate distinction between new primary tumors and metastases is critical. Comprehensive pathological evaluation, including immunohistochemistry and molecular profiling, combined with detailed imaging and, when necessary, molecular analyses to confirm clonal origin, is essential. Misclassification may lead to suboptimal treatment, emphasizing the importance of multidisciplinary review. Management should be individualized, considering tumor biology, stage, patient comorbidities, and potential treatment interactions. Structured follow-up and targeted surveillance, particularly during the first three years post-diagnosis, are key to early detection and improved outcomes.

In this retrospective, single-center study, we analyzed patients with MPMTs, of which, at least one malignancy originated from the digestive tract or its accessory organs. The primary objective was to compare overall survival between patients with synchronous versus metachronous MPMTs according to the SEER criteria. Secondary objectives included identifying clinical predictors of survival and characterizing the spectrum of tumor associations observed in our tertiary referral center.

## 2. Materials and Methods

### 2.1. Study Design and Setting

We performed a retrospective, observational, non-randomized study of consecutive patients presenting to the Oncology and Radiotherapy Departments of Saint Nicholas Hospital (Pitești, Romania) from January 2020 through June 2023. The study received approval from the Ethics Committee of Saint Nicholas Hospital; informed consent was obtained from all participants.

### 2.2. Patients and Definitions

**Figure 1:**
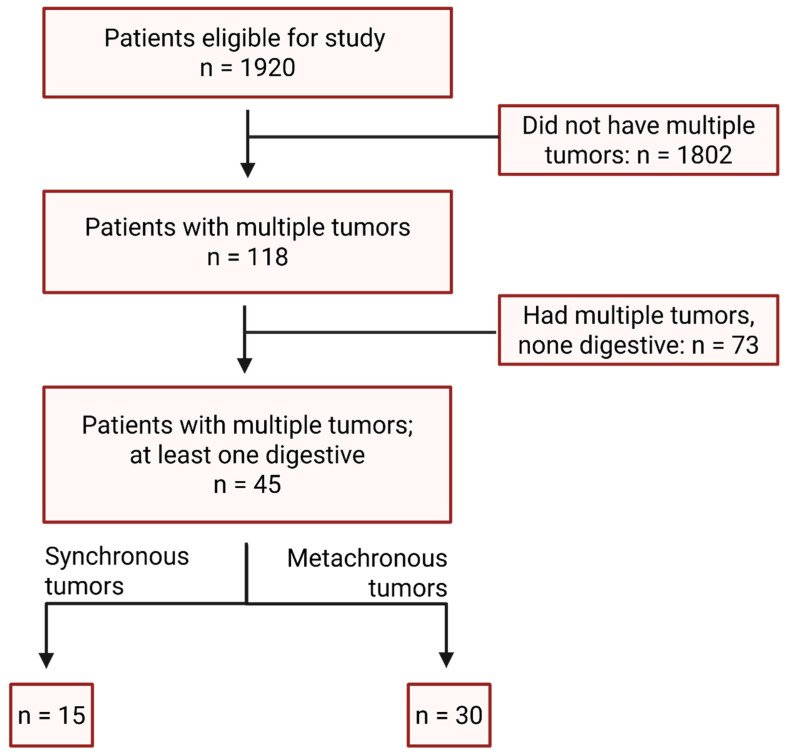
Flowchart of patients included in the study.

The inclusion criteria were (1) histopathologically confirmed malignant neoplasms, of which, at least one primary originated in the digestive tract or accessory organs. The digestive tract locations included in the study are as follows: esophagus, stomach, small intestine, large intestine (vermiform appendix, colon, rectum), anal canal; the accessory organs of the digestive tract included the pancreas, liver, bile ducts (intrahepatic bile ducts, extrahepatic bile ducts, gallbladder). Due to histopathological and therapeutic differences, the anatomical locations oral cavity, pharynx, and salivary glands were not classified as belonging to the digestive tract. (2) The patient had to have received at least one oncological diagnosis during the study period. The exclusion criteria included age < 18 years, lack of histopathological confirmation, diagnoses established post-mortem, participation in clinical trials, and cases where a second lesion was suspected to be metastatic disease rather than a distinct primary. A flowchart of patients included in the study can be found in Figure 1.

Synchronous multiple primary malignancies were defined as tumors diagnosed within two months of each other, whereas metachronous malignancies were diagnosed more than two months apart as per the SEER rules. Survival analyses were performed from the date of diagnosis of the first malignancy, for consistency with previous literature. We acknowledge that this approach may introduce lead-time and immortal-time bias, since patients who develop metachronous tumors must survive long enough after their first diagnosis to be classified as such. The TNM system provided by AJCC was used for staging oncological conditions.

Lesions were considered separate primary malignancies when clinical, radiologic, and histopathological findings supported an independent origin rather than metastatic spread. In practice, tumors located in different organs or with clearly different histologic subtypes were classified as multiple primaries. For cases with similar histology, the distinction relied on imaging features (separate organ of origin, absence of direct extension), discordant TNM stages, and multidisciplinary team consensus. Cases judged more consistent with metastatic disease were excluded according to the study criteria. Because molecular analyses were not systematically performed, a small risk of misclassification cannot be excluded, as also acknowledged in the Limitations section.

### 2.3. Data Collection

Patient records were reviewed to extract demographics (age, sex), risk factors (smoking, alcohol), tumor characteristics (histology, grade, AJCC TNM stage), treatments delivered (surgery, systemic therapy, radiotherapy), intent (curative or palliative) for each tumor, and follow-up status. For patients with three primaries, data for all tumors were captured.

### 2.4. Outcomes and Statistical Analysis

All statistical analyses were conducted using the R programming language, version 4.5.0 (R Core Team, ver. 2025). The primary endpoint was overall survival (OS), calculated from the date of diagnosis of the index tumor (first malignancy diagnosed during the study window or the first of the pair) to death from any cause or last follow-up. Survival distributions were estimated using Kaplan–Meier methods and compared with log-rank tests. Because some survival curves were not fully matured, restricted mean survival time (RMST) complemented median survival estimates. Given the limited number of outcome events, we adopted a parsimonious modeling strategy. Variables with *p* < 0.10 in univariable Cox regression were entered into a multivariable model. The prespecified covariates considered were synchronous versus metachronous presentation, primary tumor nodal stage (N0–N1 vs. N2–N3), and treatment intent for the primary tumor (curative vs. palliative). Because of the limited number of events (*n* = 17), the model was intentionally restricted to a small set of clinically relevant variables to avoid overfitting. The final model therefore included those covariates that showed the strongest univariable association and clinical relevance. Multicollinearity among variables was assessed using variance inflation factors (VIFs); values greater than 4 were considered indicative of meaningful collinearity, and none exceeded this threshold. Adjusted hazard ratios (HR) and 95% confidence intervals (CI) were reported for all included variables.

## 3. Results

### 3.1. Cohort Assembly and Baseline Characteristics

Between January 2020 and June 2023, 1920 patients with oncological diagnoses were admitted to the oncology and radiotherapy services; 118 (6.14%) had synchronous and/or metachronous MPMTs. Of these, 45 patients (38.1% of the MPMT cohort; 2.34% of all oncologic admissions) met inclusion criteria by having at least one tumor of digestive origin and a diagnosis established during the study window.

Of the 45 included patients, 25 (55.6%) were male and 20 (44.4%) were female (male:female ratio of 1.25). The age at diagnosis ranged from 36 to 86 years. For synchronous MPMTs (*n* = 15) average age at diagnosis was 66.3 years. For metachronous MPMTs (*n* = 30) the mean age at the first diagnosis was 61.9 years and 68.8 years at the time of diagnosis of the second tumor. Median follow-up for the cohort was 35.9 months. The average period between the diagnosis of the first malignant tumor and the second malignant tumor, in the case of metachronous malignant tumors, was 5 years for men and 9.2 years for women. Detailed demographic and clinical data are provided in Table 1.

Two tumors were present in 41 patients (91.1%) and three tumors in 4 patients (8.9%). Adenocarcinoma was the most frequent histology (*n* = 70; 74.46%). Fifteen patients (33.3%) had both primaries originating in the digestive tract. In the studied cohort, 10 patients (22.22%) presented consumption of alcohol and smoking as risk factors.

Notably, the subgroup counts were small (synchronous *n* = 15; several organ-specific pairings had very low n), which reduces the within-group precision and precludes robust organ-specific comparisons.

### 3.2. Tumor Associations

Colorectal–colorectal associations predominated in both groups (synchronous: 5/15, 33.3%; metachronous: 7/30, 21.2%). Other associations spanned genitourinary, upper GI, pancreatic and head & neck sites.

### 3.3. Treatment Intent

Overall, treatment intent differed between groups: curative intent for the first tumor was documented in 40 patients (89%) overall, but curative intent for the first tumor was recorded in only 10/15 (67%) of synchronous cases versus 30/30 (100%) of metachronous cases (*p* = 0.002). Palliative intent for first tumor was more frequent in synchronous disease (5/15, 33%). For second tumors, curative intent was recorded in 36 patients (80%) and palliative intent in 9 (20%).

### 3.4. Survival Analysis

At the last follow-up (October 2024), 17 of 45 patients (37.8%) had died. For the entire cohort, the RMST was 54.0 months and median OS was 41 months.

The RMST for synchronous MPMTs was 31.30 months (median OS ≈ 30 months) versus 68.3 months for metachronous MPMTs (median not reached within comparable follow-up). The unadjusted hazard ratio (synchronous vs. metachronous) was 2.49 (95% CI 0.95–6.50; *p* = 0.062). The Kaplan–Meier curves and RMST comparison suggest substantially worse survival for synchronous presentations, with the effect at the margin of statistical significance (*p* = 0.053 by log-rank).

The nodal stage of the primary tumor (N2–N3 vs. N0–N1) was associated with higher mortality (HR 4.37, 95% CI 1.24–15.3; *p* = 0.021). Similar but marginally non-significant effects were observed for nodal stage of the secondary tumor.

Treatment intent was a strong predictor: palliative intent for the primary tumor was associated with an HR of 20.5 (95% CI 3.68–114; *p* < 0.001), and palliative intent for the secondary tumor was associated with an HR of 10.3 (95% CI 3.05–30.5; *p* < 0.001).

**Figure 2:**
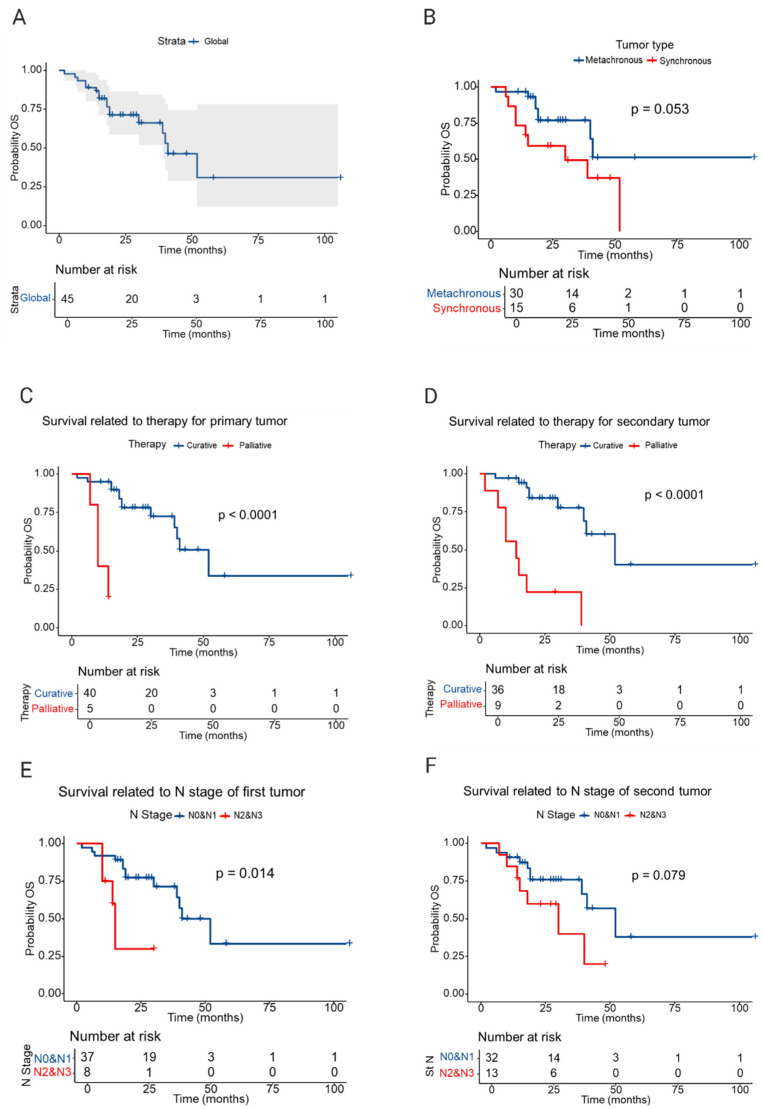
Kaplan–Meier survival curves. (**A**) Kaplan–Meier analysis of the global lot; (**B**) Graph illustrating the restricted mean survival time (RMST) for patients with synchronous and metachronous malignant tumors; (**C**) Graph illustrating the survival period depending on the type of treatment (curative or palliative) of the first malignant tumor; (**D**) Graph illustrating the survival period based on the type of treatment (curative or palliative) for the second malignant tumor; (**E**) Graph illustrating patient survival according to the N stage of the first malignant tumor; (**F**) Graph illustrating the survival of patients based on the N stage of the second malignant tumor.

RMST was lower in patients diagnosed with synchronous malignancies, treated with palliative intent, and with more advanced nodal stage (Table 2). The Kaplan–Meier plots visually reinforce the survival disadvantage in these patient subsets (Figure 2).

Table 3 summarizes the univariable Cox analyses. Table 4 reports the multivariable Cox regression results. Predictors with a univariable *p* < 0.10 (primary tumor N stage, synchronous presentation, treatment intent for the primary tumor) were entered into a backward selection model; after model reduction, primary tumor N stage (N2–N3 vs. N0–N1) remained independently associated with worse OS (adjusted HR 3.86; 95% CI 1.04–14.3; *p* = 0.044). The adjusted hazard associated with synchronous presentation was attenuated (adjusted HR 2.22, 95% CI 0.84–5.86, *p* = 0.10) and did not reach statistical significance in the final, parsimonious model (Table 4).

## 4. Discussion

The global burden of MPMTs appears to be increasing, partly due to rising life expectancy and the success of modern oncologic treatments that prolong survival after a first cancer [11]. Population-based registries have reported that the cumulative risk of developing a second primary malignancy may exceed 10% within 10 years of an initial diagnosis, especially in patients treated at younger ages [12,13]. Lifestyle-related exposures such as tobacco use, alcohol consumption, and obesity further amplify the risk of multiple primaries, particularly in the digestive tract [12,14]. These observations underscore that MPMTs are not a rare phenomenon but a growing challenge in routine clinical practice.

In addition to environmental factors, inherited cancer predisposition syndromes significantly contribute to the occurrence of MPMTs. Germline mutations in DNA mismatch repair genes are well established in Lynch syndrome, leading to excess colorectal, gastric, and endometrial cancers [15]. Similarly, germline APC mutations in familial adenomatous polyposis confer near-universal risk of colorectal neoplasia as well as duodenal and gastric tumors [16]. BRCA1/2 carriers, although more widely associated with breast and ovarian cancers, have also been shown to carry elevated risks for gastrointestinal primaries [17,18]. The identification of such syndromes is clinically relevant because cascade testing of family members and tailored surveillance can mitigate long-term mortality.

We hypothesized that host immune surveillance could influence the timing of second primaries (synchronous vs. metachronous). Emerging evidence suggests that host immune function may partly determine whether second primaries appear synchronously or metachronously. Chronic immunosuppression, whether due to solid organ transplantation, autoimmune disease therapy, or HIV infection, is associated with higher rates of multiple malignancies [19,20]. In these populations, the risk of synchronous presentation may be increased due to the inability of the immune system to contain multiple neoplastic clones simultaneously. Conversely, robust immune surveillance may delay or prevent the emergence of additional tumors, which could explain why some metachronous cases remain indolent for many years. The advent of immunotherapy has opened new questions about whether checkpoint blockade might also reduce the risk of future primaries by restoring immune recognition of early neoplastic clones, though definitive data remain lacking [21]. However, our dataset contained no immunologic or tumour-immune profiling data. Thus, any discussion of immune mechanisms or immunotherapy implications is speculative and should be regarded as hypothesis-forming. Future studies integrating immune phenotyping or tumour microenvironment analyses are needed before drawing conclusions about immunologic causality or therapeutic implications.

Therapies used for the first malignancy can themselves predispose to additional cancers. Radiation-induced sarcomas and leukemias are well-documented late effects, and certain chemotherapy agents such as alkylating agents or topoisomerase inhibitors carry established leukemogenic potential [22,23]. In gastrointestinal oncology, prolonged use of platinum-based regimens and pelvic irradiation have both been linked to higher risk of secondary tumors [24]. Distinguishing true therapy-related primaries from sporadic events is important for counseling patients and tailoring long-term surveillance.

Beyond biology, health system factors also shape outcomes in MPMTs. Access to regular follow-up, availability of high-quality imaging, and coordination within multidisciplinary teams strongly influence whether subsequent primaries are detected early enough for curative intervention [25]. Survivorship programs increasingly recognize the need for dedicated pathways for patients at risk of multiple primaries, integrating not only oncologists but also genetic counselors, psycho-oncology support, and primary care physicians. In low- and middle-income countries, limited resources may delay detection of second malignancies, contributing to poorer outcomes compared with high-income settings [26].

The rising prevalence of MPMTs has major implications for cancer control strategies. Screening programs for colorectal, gastric, and hepatocellular carcinoma may incidentally detect second primaries, but current guidelines rarely account for the elevated risk in survivors of prior cancers [27,28]. Incorporating prior cancer history into risk prediction models could optimize screening intensity and improve cost-effectiveness. At a policy level, increasing awareness of MPMTs among clinicians and patients alike is necessary to ensure timely presentation and avoid misinterpretation of new lesions as metastases.

In this context, we analyzed 45 patients with at least one digestive tract primary tumor to assess survival outcomes and prognostic factors. The percentage of patients diagnosed with multiple malignant tumors and the ratio of diagnoses in men and women in the studied group are similar to those observed by other authors [4]. Our findings are broadly consistent with prior reports that synchronous multiple primary malignant tumors (MPMTs) are associated with inferior outcomes compared with metachronous presentations. For example, Lv et al. found that synchronous MPMTs, particularly those involving the digestive tract, had significantly reduced survival compared to metachronous cases, with the interval between tumors being a key prognostic factor (3-year OS 20.9% vs. 58.0%) [8]. Similarly, Baba et al. demonstrated that synchronous multiple primary cancers in esophageal cancer patients independently predicted poorer long-term survival, with a multivariate HR of 1.61 (95% CI 1.08–2.36) [29]. Jiao et al. reported median survival times of 3.8 years for those with synchronous and 17.3 years for those with metachronous MPMTs, further supporting the survival disadvantage of synchronous presentations [30]. In colorectal cancer, Fan et al. found 5-year overall survival rates of 68.6% for synchronous versus 81.9% for metachronous cases, with synchronous disease associated with more advanced stage and adverse molecular features [31]. Kollias et al. confirmed similar patterns in a breast cancer cohort [32]. Our data in digestive tract primaries demonstrated a 2.5-fold increased hazard of death for synchronous versus metachronous disease, in line with the general impression that synchronous primaries complicate management and are often detected at a more advanced stage.

Several mechanisms may explain this pattern. Synchronous tumors are frequently diagnosed in the setting of already symptomatic disease, often when staging investigations reveal an unexpected second lesion. This may mean both primaries are detected later in their natural history. In addition, treatment sequencing is particularly challenging: oncologists must decide which tumor should be treated first, whether therapies can be given concurrently, and how to balance toxicity across regimens. These complexities increase the likelihood that curative therapy cannot be delivered to both tumors, which is reflected in our cohort by the higher proportion of palliative intent in the synchronous group.

By contrast, metachronous tumors typically arise years after the first diagnosis, often during structured follow-up when surveillance imaging or endoscopy allows for earlier detection. In our cohort, the average interval between the first and second diagnoses in metachronous cases was 5 years for men and over 9 years for women. This window may allow patients to recover from prior therapies, undergo curative resections, and remain candidates for multimodal treatment. The survival advantage seen in metachronous patients may therefore relate both to the earlier stage at detection and to a greater feasibility of curative treatment.

However, contradictory findings have been reported in certain tumor types. Bugter et al. noted that in head and neck cancers, patients with metachronous tumors could in fact have surprisingly favorable survival (5-year OS: 85% vs. 25% for synchronous cases), emphasizing that the prognostic effect of timing is context- and disease-dependent [33]. Jiang et al. performed a meta-analysis in multiple primary lung cancers and found that while overall survival was inferior for synchronous versus metachronous cases when measured from the first tumor, there was no significant difference in survival when measured from the second tumor (HR 1.19, 95% CI 0.86–1.66; *p* = 0.29) [34]. Komatsu et al. observed that metachronous MPMT patients diagnosed more than five years after NSCLC resection did not differ in prognosis from those with single primary NSCLC, suggesting that the timing and interval between tumors may modulate survival impact [35]. In melanoma, Sarver et al. found improved survival for patients with second primary melanomas compared to single primaries [36], while Karapetyan et al. reported worse outcomes for multiple versus single primary melanomas, highlighting disease-specific variability [37].

While the present cohort and most published series indicate poorer outcomes for synchronous compared with metachronous MPMTs, several studies have shown that this difference may diminish or become statistically non-significant in certain organs. This variability likely reflects differences in tumor biology, treatment feasibility, and patterns of surveillance. In organs where synchronous tumors show worse outcomes, such as the aerodigestive or digestive tract, simultaneous lesions often arise through field cancerization or shared carcinogenic exposure, producing biologically aggressive, multifocal disease that limits curative treatment. In contrast, in organs where the survival difference may be reduced or non-significant, such as the lung or skin, the later appearance of a new primary may indicate persistent carcinogenic exposure, accumulated genetic damage, or impaired immune surveillance, reflecting an intrinsically high-risk host background. Differences in available therapies and detection timing further modify this relationship: synchronous tumors may restrict surgical options, whereas metachronous lesions, although temporally separated, may develop after systemic therapy or radiotherapy, in a setting of compromised organ reserve. These elements together may explain why the survival gap between synchronous and metachronous tumors varies across organ systems and sometimes loses statistical significance.

In summary, the current findings of worse survival in synchronous versus metachronous MPMTs are strongly supported by the majority of studies in digestive and other solid tumors, though exceptions exist in specific cancer types and when accounting for tumor interval and biology.

Additional studies in lymphoma and other solid tumors reinforce the importance of tumor burden, histology, and treatment sequence in modulating survival outcomes for synchronous MPMTs. The literature consistently emphasizes the need for individualized management and careful surveillance, particularly in patients at risk for synchronous presentations.

Our analysis also highlighted nodal burden as a particularly strong prognostic factor. Patients with N2–N3 disease at the time of primary tumor diagnosis had a nearly fourfold increased risk of death compared to those with N0–N1 disease, and this association persisted in multivariable modeling. This finding is important because nodal stage is a potentially modifiable determinant through early diagnosis and appropriate locoregional control. It also suggests that careful staging at presentation, including the use of advanced imaging and nodal sampling, remains essential not only for guiding treatment but also for long-term risk stratification.

Treatment intent emerged as another dominant driver of survival. Patients treated with curative intent had approximately double the restricted mean survival time compared to those treated palliatively, regardless of whether the tumor was the first or second malignancy. While palliative treatment intent was strongly associated with worse survival, this variable likely functions predominantly as a mediator or consequence of advanced and incurable disease rather than an independent causal predictor. In other words, advanced stage and nodal burden influence the decision to select palliative intent, which in turn determines survival. Given our observational data we cannot infer causal direction. In future work, time-dependent models or causal mediation analyses in larger datasets would be preferable to clarify these pathways.

Our results should also be considered in the context of surveillance strategies. Forty percent of patients developed a new malignancy during follow-up, a proportion that reinforces the importance of structured long-term monitoring. Current practice often limits surveillance intensity to five years after initial therapy, but our data suggest that patients with digestive tract primaries remain at risk well beyond that period. Tailoring surveillance protocols to individual risk factors such as nodal stage, lifestyle exposures, and family history may improve early detection of subsequent primaries. In practical terms, patients with advanced nodal disease or other high-risk features may benefit from closer clinical and imaging follow-up during the first few years after treatment, while maintaining vigilance in the longer term. For colorectal primaries, adherence to guideline-based colonoscopic surveillance remains essential and could be individualized in selected high-risk cases. Although our findings do not justify formal guideline modification, they highlight the need for a proactive, multidisciplinary approach and a low threshold for investigating new symptoms in cancer survivors.

This study has several limitations. Its retrospective nature makes it vulnerable to selection and information bias, and the small sample size (*n* = 45, 17 events) limits the statistical power, particularly for subgroup analyses. Several subgroup strata contained very low counts (some organ pairings *n* < 5), which reduces the internal validity and increases the risk of chance findings. Although we attempted to control for key prognostic variables, residual confounding is possible. The single-center setting may reduce generalizability, and molecular data were unavailable in most cases, preventing us from distinguishing sporadic second primaries from therapy-related or clonally related lesions. Classification between new primaries and metastatic disease relied mainly on histological and imaging criteria. The comparison between synchronous and metachronous cases must be interpreted with caution. Because patients need to survive long enough to develop a metachronous second primary, the analysis is subject to immortal-time bias. Differences in tumor stage and treatment intent may also partially explain the observed survival disparities. We therefore present these analyses as exploratory and hypothesis-generating as our findings indicate association rather than causation. Nevertheless, our cohort provides valuable real-world insights into a relatively underexplored clinical scenario.

Future work should focus on prospective, multicenter registries that integrate molecular characterization, treatment sequencing, toxicity, and patient-reported outcomes. Such efforts would allow for robust modeling of prognostic factors and identification of optimal therapeutic strategies, particularly for synchronous primaries where evidence remains scarce. Randomized trials may be infeasible, but large observational datasets and international collaborations can help fill this gap.

In summary, synchronous presentation was associated with inferior unadjusted survival, but nodal stage and the feasibility of curative therapy were the most decisive predictors of outcome. Given the small sample size and retrospective design, our findings are associative and should be considered preliminary. They support the need for dedicated prospective registries and molecular characterization to validate prognostic markers and refine surveillance strategies for patients at risk of multiple primaries.

## 5. Conclusions

In our single-center series, synchronous MPMTs showed a tendency toward shorter survival compared with metachronous cases, but the difference did not reach statistical significance. Advanced nodal stage and palliative treatment intent were the dominant independent factors associated with poor outcome. These observations support vigilant long-term surveillance after treatment of digestive tract cancers and highlight the need for individualized, multidisciplinary management when multiple primaries are identified. Given the small sample size and retrospective design, the results should be regarded as preliminary and hypothesis-generating. Validation in larger, multicenter cohorts with integrated molecular data is required before firm clinical recommendations can be made.

## Figures and Tables

**Table 1:** Demographic and clinical data of patients with multiple malignant tumors.

Clinical Variable	Synchronous Malignant Tumors	Metachronous Malignant Tumors
Average age at diagnosis (years)		
First malignant tumor	66.28	61.9
Second malignant tumor		68.8
Sex		
Male	9	16
Female	5	15
Tumor grade		
G1	6 (18.75%)	18 (29.5%)
G2	21 (65.62%)	35 (57.37%)
G3	5 (15.62%)	8 (13.11%)
TNM stage		
I	2 (7.4%)	10 (15.1%)
II	4 (14.8%)	21 (31.8%)
III	16 (55.5%)	27 (40.9%)
IV	5 (18.51%)	8 (12.1%)
Treatment intent		
Curative		
First malignant tumor	10 (32.25%)	30 (47.61%)
Second malignant tumor	10 (32.25%)	26 (41.26%)
Third malignant tumor	1 (3.22%)	2 (3.17%)
Palliative		
First malignant tumor	5 (16.12%)	0 (0.00%)
Second malignant tumor	5 (16.12%)	4 (6.34%)
Third malignant tumor	0 (0.00%)	1 (1.58%)
Patients diagnosed with a second malignant tumor during follow-up after treatment of the first malignant tumor	2	16

G (grade), TNM (tumor-node metastasis).

**Table 2:** Survival of patients with multiple malignant tumors based on tumors type, treatment and nodal stage.

Clinical Variable	Deaths N (%)	RMST (Months)	Median Survival
The entire lot	17/45 (37.77)	54.00	41.00
Tumor type			
Metachronous	8/30 (26.66)	68.30	N/A (40 at N/A)
Synchronous	9/15 (60)	31.30	30 (14 at N/A)
Treatment T1			
Curative	13/40 (32.50)	58.20	52.00
Palliative	4/5 (80.00)	29.40	10.00
Treatment T2			
Curative	9/36	64.60	52.00
Palliative	8/9 (88.88)	17.10	14.00
Nodal stage T1			
0–I	13/37 (35.13)	57.30	41.00
II–III	4/8 (50.00)	40.90	14.00
Nodal stage T2			
0–I	10/32 (31.25)	60.90	52
II–III	7/13 (53.84)	40.30	30

**Table 3:** Clinical factors associated with survival in patients diagnosed with synchronous and metachronous malignant tumors.

Predictor	N	Deaths N	HR (95% CI)	*p*-Value
Age				
T1	45	17		
T2	45	17	1.03 (0.97 to 1.10)	0.382
Sex				
Female	20	8		
Male	25	9	0.87 (0.33 to 2.27)	0.768
Tumor type				
Metachronous	30	8	-	
Synchronous	15	9	2.49 (0.95 to 6.50)	0.062
Nodal stage T1				
0	37	13	-	
II–III	8	4	4.37 (1.24 to 15.3)	0.021
Nodal stage T2				
0				
II–III	13	7	-	-
Grade T1				
G1	17	7	-	-
G2	25	9	1.03 (0.38 to 2.79)	0.951
G3	3	1	1.17 (0.14 to 9.70)	0.887
Grade T2				
G1	8	3	-	-
G2	27	10	0.80 (0.22 to 2.97)	0.741
G3	10	4	0.94 (0.21 to 4.30)	0.936
Treatment intent T1				
Curative	40	13	-	-
Palliative	5	4	20.5 (3.68 to 114)	<0.001
Treatment intent T2				
Curative	36	9		
Palliative	9	8	10.3 (3.051 to 30.5)	<0.001

**Table 4:** Multivariable Cox proportional hazards regression for overall survival. Model building stated in Methods. VIFs were <4 for included covariates.

Predictor	N	Deaths	HR (95% CI)	*p*-Value	VIF
Tumor type					1.0
Metachronous	30	8	-		
Synchronous	15	9	2.22 (0.84 to 5.86)	0.1	
Nodal stage T1					
0–I	37	13	-		
II–III	8	4	3.86 (1.04 to 14.3)	0.044	

## Data Availability

The original contributions presented in this study are included in the article. Further inquiries can be directed to the corresponding author.
